# Understanding Self-Guided Web-Based Educational Interventions for Patients With Chronic Health Conditions: Systematic Review of Intervention Features and Adherence

**DOI:** 10.2196/18355

**Published:** 2020-08-13

**Authors:** Li Feng Xie, Alexandra Itzkovitz, Amelie Roy-Fleming, Deborah Da Costa, Anne-Sophie Brazeau

**Affiliations:** 1 School of Human Nutrition McGill University Sainte-Anne-de-Bellevue, QC Canada; 2 Department of Medicine McGill University Montreal, QC Canada; 3 Montreal Diabetes Research Center Montreal, QC Canada

**Keywords:** chronic disease, online learning, self-management, mobile phone

## Abstract

**Background:**

Chronic diseases contribute to 71% of deaths worldwide every year, and an estimated 15 million people between the ages of 30 and 69 years die mainly because of cardiovascular disease, cancer, chronic respiratory diseases, or diabetes. Web-based educational interventions may facilitate disease management. These are also considered to be a flexible and low-cost method to deliver tailored information to patients. Previous studies concluded that the implementation of different features and the degree of adherence to the intervention are key factors in determining the success of the intervention. However, limited research has been conducted to understand the acceptability of specific features and user adherence to self-guided web interventions.

**Objective:**

This systematic review aims to understand how web-based intervention features are evaluated, to investigate their acceptability, and to describe how adherence to web-based self-guided interventions is defined and measured.

**Methods:**

Studies published on self-guided web-based educational interventions for people (≥14 years old) with chronic health conditions published between January 2005 and June 2020 were reviewed following the PRISMA (Preferred Reporting Items for Systematic Reviews and Meta-Analyses) Statement protocol. The search was performed using the PubMed, Cochrane Library, and EMBASE (Excerpta Medica dataBASE) databases; the reference lists of the selected articles were also reviewed. The comparison of the interventions and analysis of the features were based on the published content from the selected articles.

**Results:**

A total of 20 studies were included. Seven principal features were identified, with goal setting, self-monitoring, and feedback being the most frequently used. The acceptability of the features was measured based on the comments collected from users, their association with clinical outcomes, or device adherence. The use of quizzes was positively reported by participants. Self-monitoring, goal setting, feedback, and discussion forums yielded mixed results. The negative acceptability was related to the choice of the discussion topic, lack of face-to-face contact, and technical issues. This review shows that the evaluation of adherence to educational interventions was inconsistent among the studies, limiting comparisons. A clear definition of adherence to an intervention is lacking.

**Conclusions:**

Although limited information was available, it appears that features related to interaction and personalization are important for improving clinical outcomes and users’ experience. When designing web-based interventions, the selection of features should be based on the targeted population’s needs, the balance between positive and negative impacts of having human involvement in the intervention, and the reduction of technical barriers. There is a lack of consensus on the method of evaluating adherence to an intervention. Both investigations of the acceptability features and adherence should be considered when designing and evaluating web-based interventions. A proof-of-concept or pilot study would be useful for establishing the required level of engagement needed to define adherence.

## Introduction

### Background

Chronic diseases contribute to 71% of deaths worldwide every year, which corresponds to 41 million deaths per year. It has been estimated that among these deaths, 15 million people between the ages of 30 and 69 years die mainly because of cardiovascular disease, cancer, chronic respiratory diseases, or diabetes [1]. Apart from mortality, the consequences of these chronic diseases include a decrease in the quality of life [2,3] and an economic burden for both households and countries [4-6]. The use of information and communication technology for health-related purposes has the potential to mitigate these consequences by offering numerous benefits for disease management, such as facilitating access to health information and helping to increase the understanding of the disease [7]. It is also considered a flexible, low-cost method for patients to obtain information in comparison with face-to-face education sessions [8]. Web-based interventions are an example of information and communication technology that has the potential to educate people living with a specific chronic disease condition and can help to improve their self-care over the long term through education and peer support [8,9]. These web-based interventions can be in a guided format by including features such as electronic counseling (e-counseling) and long-distance monitoring by health care professionals (HCPs) [10] or can be self-guided, defined in this paper as an absence of individual or face-to-face contact between HCPs and the users. Previous studies have investigated the integration of various features (eg, reminders and opportunities for social support) and the design of these web-based interventions. They concluded that the implementation of specific features and degree of adherence to the intervention are key factors in determining their success [11,12]. However, these studies do not distinguish between interventions with one-on-one or in-person contact among users with (guided) and without (self-guided) an HCP. As contact with HCPs or e-consultations can lead to a higher cost per usage and decrease the accessibility of the intervention [13], it is important to understand the inclusion of specific features and evaluation of adherence to these self-guided interventions.

The definition and measurement of adherence to self-guided interventions are still subject to debate [14,15]. Adherence is defined by the World Health Organization as “the extent to which a person’s behaviour – taking medication, following a diet, and/or executing lifestyle changes, corresponds with agreed recommendations from a health care provider” [16]. However, this definition is not adapted in the context of information and communication technology; there is no prescribed dosage that users of specific web-based interventions should be using to have the expected behavioral change [12]. The difficulty in defining adherence to web-based self-guided interventions is further accentuated by the differences in which they have been measured across studies with the use of parameters, such as the number of log-ins, the content viewed, and/or the time spent on the intervention [14].

### Objective

A deeper understanding of previously published evaluations of self-guided educational interventions is required. The goals of this systematic review are to investigate how web-based intervention features are evaluated to determine their acceptability and to explore how adherence to web-based self-guided interventions are defined and measured. An understanding of the specific features and standardization of the definition of adherence to web-based self-guided interventions can help increase their efficacy and help to develop future web-based interventions for disease management.

## Methods

### Design and Search Strategies

To achieve these objectives, a systematic review of studies investigating the acceptability of the included features in web-based educational interventions on chronic health conditions was conducted based on the Preferred Reporting Items for Systematic Reviews and Meta-Analyses framework [17]. For this review, chronic health conditions also include chronic diseases. Nine chronic health conditions were selected from a list of common chronic diseases in Canada [18]. The selection of these studies was related to the implication of a web-based educational intervention on patients’ self-management and their commonality across different age groups. Cancer and mental illness were excluded from this systematic review because of the broad variety of disease and treatment methods [19-21]. The selected categories were defined as follows: (1) arthritis, (2) celiac disease, (3) epilepsy, (4) inflammatory bowel disease (including Crohn disease and ulcerative colitis), (5) metabolic disorders (including hypertension, dyslipidemia, atherosclerosis, heart failure, gestational diabetes mellitus [GDM], and type 1 and type 2 diabetes mellitus), (6) multiple sclerosis, (7) overweight and obesity, (8) respiratory disease (including chronic respiratory disease, asthma, and chronic obstructive pulmonary disease [COPD]), and (9) kidney diseases (including end-stage renal disease and nephritis).

The search method was elaborated with the help of a librarian. The PubMed, Cochrane Library, and EMBASE (Excerpta Medica dataBASE) databases were used to ensure that all articles related to the topic were covered. Keywords (Textbox 1), derived from Medical Subject Headings (MeSH), were searched in the titles or abstracts. The search combined each medical condition with the web-based, education, and intervention terms. A full list of the search methods is included in Multimedia Appendix 1. If the clinical trial protocol was available, the corresponding author’s name and the study title were further searched on these databases to find the relevant publications. The reference lists of the selected articles were also screened to capture potential articles. The screening and selection of the articles were performed independently by 2 reviewers (LFX and AI), and consensus was reached through a discussion to ensure agreeability. A third researcher (ASB) was consulted for a nonunanimous discussion for the selection of the articles.

**Keywords used for the article searches for different categories.**

Web-based

- “social media” OR Internet OR “web based” OR web OR online

Education

- “distance education” OR education OR “patient education” OR teaching

Intervention

- learning OR intervention OR treatment OR program OR “Program development” OR platform

Arthritis

- arthritis

Celiac disease

- celiac

Epilepsy

- epilepsy

Inflammatory bowel disease

- IBD OR “inflammatory bowel disease” or “crohn disease” or “ulcerative colitis”

Metabolic disorders

- CVD OR hypertension OR diabetes OR “diabetes mellitus” OR “diabetes insipidus” OR “gestational diabetes” OR “type 2 diabetes mellitus” OR “type 1 diabetes mellitus” OR “Juvenile diabetes” OR “heart failure” OR atherosclerosis OR dyslipidemia OR “Cardiovascular disease”

Multiple sclerosis

- “multiple sclerosis”

Obesity

- “pediatric obesity” OR “abdominal obesity” OR “morbid obesity” OR “obesity management” OR “Abdominal obesity” OR “metabolic syndrome” OR “overweight” OR “metabolic syndrome” OR “weight reduction program”

Respiratory disease

- “respiratory disease” or “respiratory tract disease” or “respiratory disorder” or “asthma” or “chronic respiratory disease” or “copd” or “chronic obstructive pulmonary disease”

Kidney disease

- “chronic kidney disease” or “chronic renal insufficiency” or “kidney disease” or “chronic kidney failure” or “diabetic nephropathies” or “esrd” or “end stage renal disease” or “nephritis”

### Study Selection

Inclusion criteria were as follows: (1) the study included a web-based educational intervention designed for people living with this health condition (eg, transfer of knowledge to this population), (2) the intervention aimed to improve clinical outcomes defined as the result of a health care intervention, which includes a change in clinical laboratory values (eg, level of blood glucose, blood lipid profile), lifestyle behavior (eg, improvement in eating habits and level of physical activity), use of health care system (eg, use of emergency department and length of hospitalization), and quality of life [22] related to an existing chronic health condition, (3) no in-person or one-to-one contact with an HCP within the intervention, (4) only contacted the research team for technical support or an introductory meeting during the intervention (to ensure the pragmatism of the study results [23] and limit the impact of these contacts on the adherence to the intervention), (5) the included population is ≥14 years old (age cutoff where people can make their own health care decisions in Quebec, Canada [24]), (6) the articles (published or in-press, to have a full portrait of the intervention and have peer-reviewed evidence) were published between January 1, 2005, and June 15, 2020, in English or in French, (7) the articles are fully available to the researchers, and (8) no restriction on the design of the study but only original research was included.

Studies corresponding to any of the following criteria were excluded from this systematic review: (1) the intervention is for family members or HCPs only, (2) the intervention has only a purpose of prevention/assessment/screening aftercare, (3) the web-based intervention included a live session or personalized e-counseling, (4) the intervention consisted of only emails, discussion forums, and/or recording functions, (5) the study explicitly stated an inclusion of participants with severe depression, and (6) the primary target outcome was related to mental health.

### Data Extraction and Analysis

For each study, the following information was collected and compared: the year of publication, country where the study took place, study design, targeted chronic health conditions, primary clinical outcomes, age group of the population, sample size, intervention given to the experimental and control groups, and length of the intervention.

In this study, a feature is defined as any functionality within a web-based educational intervention other than text-based educational modules, supporting users to have a better learning or navigation experience or to improve clinical outcomes. The term feature and functionality are used interchangeably for this review. Both analyses of acceptability of the features and adherence to the intervention were based on reported information contained in the articles or the complete protocol cited from the selected articles. The method for evaluating features and their acceptability on the outcomes of the intervention are discussed. The measurement and criteria used to evaluate adherence to the intervention were collected and compared between studies.

All the data were collected from information within the articles, the related published supplementary documents, or the cited references. If >1 article reported the same intervention and outcomes but had different sample sizes, then articles stating results of the acceptability of the features or adherence to the intervention were reported. If none or all the articles reported these details, the latest publication was analyzed. However, information related to the acceptability of the features and adherence was collected from all related articles. If 2 interventions within the same study correspond to the inclusion criteria of this review, the intervention with the highest number of features was analyzed. The data from each study were then grouped into themes. EndNote X9.2 for Macintosh was used to regroup the articles.

## Results

### Study Selection

**Figure 1:**
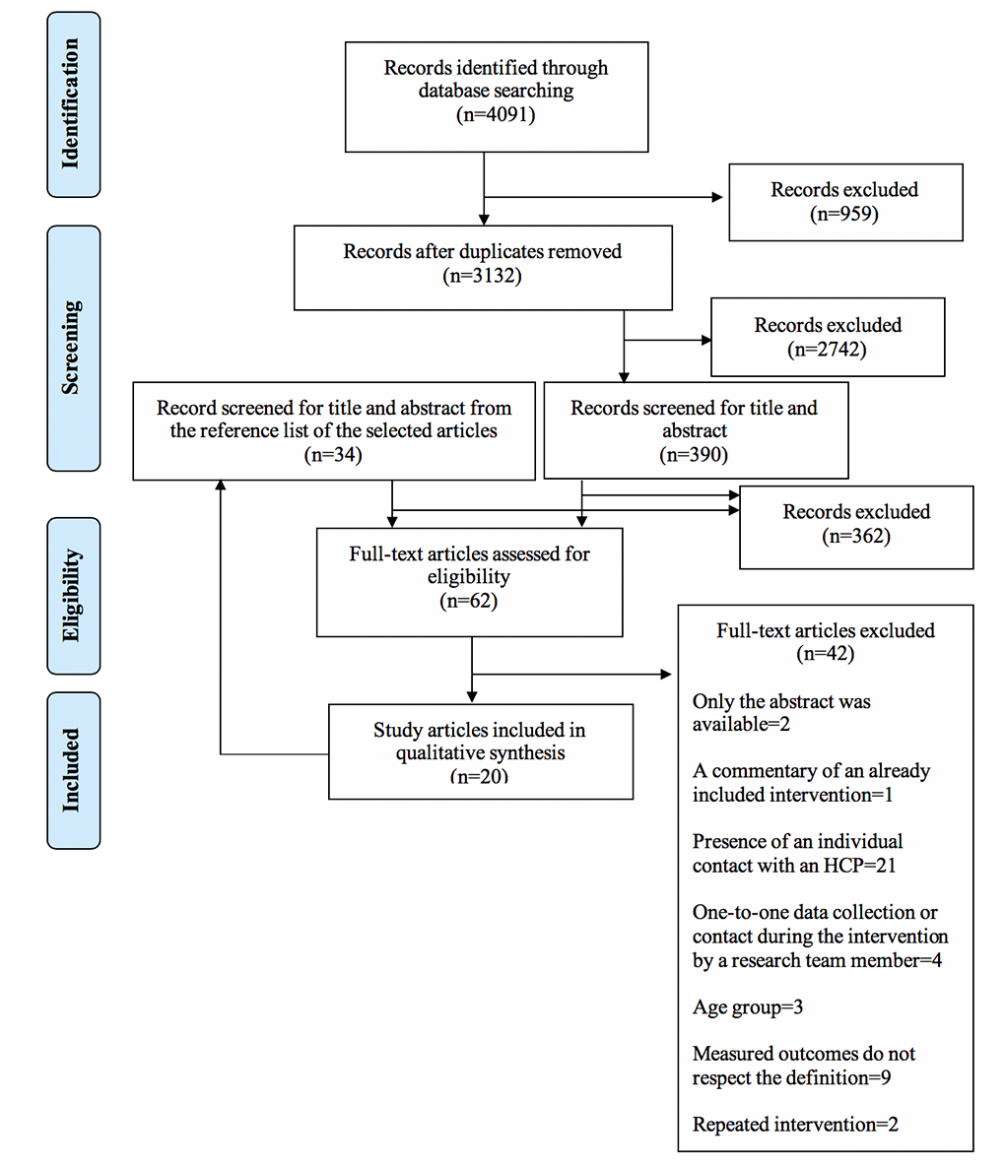
Study selection.

The searches on the PubMed, Cochrane Library, and EMBASE databases resulted in 4091 potentially eligible articles (Figure 1). The titles and abstracts were reviewed, resulting in 390 articles. The titles and abstracts of potential articles from the reference list of the selected articles were also reviewed (n=34). After reading the full articles, 20 studies were selected.

### Study Characteristics

The study characteristics are presented in Table 1. The identified articles included 6 areas of chronic health conditions: arthritis (n=1) [25], celiac disease (n=1) [26], metabolic disorders other than weight issues (n=8; metabolic syndrome [27], cardiac condition [28], hypertension [29], type 2 diabetes [30-33], and GDM [34]), multiple sclerosis (n=1) [35], overweight and obesity (n=7 studies) [13,36-41], and respiratory diseases (n=2; asthma [42] and COPD [43]). No study related to epilepsy, inflammatory bowel disease, or chronic kidney disease was found. The primary clinical outcomes were mainly related to changes in weight [13,36-41]. The studies were predominantly conducted in the United States [13,27,28,31,33,39-41,43] and Australia [25,26,34,36-38,42]. All the selected studies were randomized controlled trials, except for the study by Hutchesson et al [36], which was a pre-post design, and the study by Umapathy et al [25], which had a quasi-experimental design. All selected articles were published in English.

**Table 1:** Study characteristics and description of the interventions.

Study; primary clinical outcomes	Health conditions	Study design; length of the intervention	Population, sample size	Descriptions of the interventions given to the experimental group	Descriptions of the interventions given to the control group
Bosak, 2010, United States [27]; minutes of PA^a^, energy expenditure per week	Metabolic syndrome	RCT^b^; 6 weeks	Adults ≥19 years; n=22	In-person introductory session, weekly new content, goal setting, self-monitoring, quiz, feedback (by email and after a quiz), use of persona, discussion board monitored by the PI^c^; general discussion question posted by the PI on the forum. Requested at least weekly participation in the discussion^d^ (n=12, with 57% men)	Usual care (assessment by physicians and a consultation with a dietitian); (n=10, with 80% men)
Burns, 2013, Australia [42]; asthma control, self-efficacy, QoL^e^	Asthma	RCT; 3 months	Adults with asthma ≥55 years; n=51	Six 15-min modules, reminder email to the nonresponders^d^ (with 33% men)	None
Carolan-Olah, 2019, Australia [34]; BMI, blood pressure, glycemic level	GDM^f^	RCT; ND^g^	Singleton pregnant women aged 18-45 years with recently diagnosed GDM; n=110	Standard GDM program and an additional 41-module web-based program including a one-on-one 30-min introductory session and quizzes^d^ (n=52)	Standard GDM program (1.5 hours of in-person class education given by HCPs^h^; n=58)
Hansel, 2017, France [30]; change of the dietary score	T2DM^i^	RCT; 16 weeks	Adults with T2DM and abdominal obesity, 18-75 years; n=120	4 modules, videos, hotline technical support, and feedback on the self-monitoring data and pedometer outcomes; requested at least 11 weekly log-in^d^ (n=60, with 33% men)	Usual follow-up with HCPs (n=60, with 33% men)
Hutchesson, 2016, Australia [36]; weight, BMI, WC^j^	Overweight	Pre-post design; 3 months	Women aged 18-30 years; n=26	Web-based quizzes to assess current health status (diet, exercise, weight) with email feedback report, goal setting, discussion forum monitored by a dietitian, smartphone app, email newsletters, text messages, graphic design reflecting the image of the population^d^	None
Jane, 2017, Australia [37]; weight	Obesity	RCT; 24 weeks	Adults aged 21-65 years; n=67	1. Leaflet group with pedometer: weight loss information contained in a booklet (n=23, with 9% men); 2. Facebook group with pedometer: same weight loss information within a booklet but with pages only accessible via the Facebook group. The group was monitored by the study coordinator and this person made a weekly post^d^ (n=23, with 17% men); all the groups: 30-min introductory session	Standard care following Australian dietary and physical activity guidelines (n=21, with 19% men)
Kessel, 2016, New Zealand [35]; fatigue severity and impact	MS^k^	RCT; 8-10 weeks	Adults^l^ experiencing MS fatigue; n=39	MSInvigor 8 plus: MSInvigor8 intervention with email-based support provided by a clinical psychologist for guidance and personal feedback (n=19, with 42% men)	MSInvigor8: cognitive behavior therapy–based 8 sessions with printable document, audio, and video; 25-50 min to complete; automated email reminders^d^ (n=20, with 10% men)
Kerfoot, 2017, United States [31]; HbA_1c_^m^	T2DM	RCT; 6 months	Veterans with T2DM; n=456	Team-based web game with questions related to DSME^n^ and a civic booklet about American history; other features: multiple-choice questions via email or smartphone app, same questions resent in a cycled pattern, points given for the quiz answer, feedback after the quiz, team and individual financial reward (US $100 gift certificate^d^; n=227, with 95% men)	Same game format as the intervention group but with game questions on civics and a DSME booklet (n=229, with 93% men)
Leahey, 2014, United States [13]; weight	Obesity	RCT; 3 months	Adults aged 18-70 years; n=230	Group 1: the ShapeUp Rhode Island 2011 (SURI) program plus an internet behavioral weight loss program. Included a 60-min introductory session, self-monitoring, and feedback on the progress^d^ (n=90, with 18% men); group 2: the previous program plus optional weekly face-to-face group sessions (n=94, with 14% men)	SURI program alone: team participation, self-monitoring, pedometer, newsletters, community workshops, and recognition for meeting goals (n=46, with 18% men)
Liu, 2018, Canada [29]; systolic blood pressure	HTN^o^	RCT; 4 months	Adults aged 35-74 years with HTN; n=128	1. Web expert-driven group with a prescribed weekly exercise and diet plan (n=43, with 51% men); 2. web user-driven group with weekly email where participants can choose their exercise and diet goals^d^ (n=42, with 48% men); in both groups, the same contents are under video and text format	Weekly email newsletter on HTN management only (n=43, with 57% men)
Morgan, 2011, Australia [38]; weight	Obesity	RCT; 3 months	Men aged 18-60 years; n=65	75-min face-to-face introductory session, self-monitoring, goal setting, feedback, and online forum weekly monitored by the research team^d^ (n=31)	60-min face-to-face introductory session and a weight loss program booklet (n=34)
Moy, 2016, United States [43]; HRQoL^p^	COPD^q^	RCT; 4 months	Veterans with COPD, n=239	Goal setting, self-monitoring, feedback for the self-monitoring data, reminder, discussion forum, technical support, and pedometer^d^ (n=155, with 95% men)	Pedometer with 12-month delayed access to the web intervention (n=84, with 92% men)
Noh, 2010, Korea [32]; postprandial glucose, HbA_1c_	T2DM	RCT; 6 months	Adults with T2DM aged 18-80 years; n=40	6-module program, adaptation to smartphones^d^ (n=20, with 80% men)	Same educational content in a printed booklet (n=20, with 75% men)
Richardson, 2007, United States [33]; steps	T2DM	RCT; 6 weeks	Nonpregnant adults with T2DM; n=35	Basic intervention with automated step goals based on the previous weekly total accumulated steps^d^ (n=17, with 29% men)	Basic intervention (60-min introductory session, pedometer, access to web-based educational information, tailored motivational messages, feedback for the performance) with step goals based on walking bouts >10 min with at least 60 steps per minute (n=13, with 62% men)
Rothert, 2006, United States, [39]; weight	Overweight and obesity	RCT; 6 weeks	Adult with BMI 27-40 kg/m^2^; n=286	Tailored expert system: automated personal weight management plan delivered at 1, 3, and 6 weeks of the study; reminders and choice of encouragement message via email^d^ (n=1475, with 17% men)	Information-only: standard Kaiser Permanente weight loss website (n=1378, with 13% men)
Sainsbury, 2013, Australia [26]; gluten-free diet adherence	Celiac disease	RCT; 8 weeks	Patients ≥16 years with biopsy-confirmed celiac disease (n=189, with 13% men)	Six 30-min modules^d^ (n=101)	Access to the intervention after 8 weeks of randomization (n=88)
Tate, 2006, United States, [40]; weight	Overweight and obesity	RCT; 6 months	Adults (20-55 years) with a BMI 27-40 kg/m^2^; n=122	1. Basic intervention with an additional website that includes electronic diary, message board, additional weekly reminder emails, weekly automated email feedback^d^ (n=61, with 13% men); 2. same intervention as in 1 but email feedback was given by a human counselor (n=64, with 16% men)	Basic intervention: introductory face-to-face group session, diet and energy expenditure goal, access to Slim-Fast website, meal-replacement coupon, optional web matching with another participant, weekly report, email communications (n=67, with 18% men)
Thomas, 2015, United States [41]; weight	Obesity	RCT; 3 months	Adults aged 18-70 years; n=154	60-min introductory session, video, animation, quiz, self-monitoring, weekly feedback about participant’s progress, reminders, and recognition for meeting the goals^d^ (n=15, with 20% men)	Introductory session, printable newsletters with educational information on diet and physical activity; requested at least weekly log-in (n=16, with 21% men)
Umpathy, 2015, Australia [25]; heiQ^r^	OA^s^	Quasi-experimental study; 12 months	Adults with self-assessed hip and/or knee OA; n=195	My Joint Pain: educational modules (text or video) with self-assessment tools^d^ (n=104, with 24% men)	No intervention was provided from the study (n=91, with 20% men)
Widmer, 2017 2015, United States [28,44]^t^; CV-related ED visits^u^ and rehospitalizations	Cardiac condition	RCT; 3 months	Eligible patients to a regular cardiac rehabilitation; n=80	Regular cardiac rehabilitation with digital health: 30-min introductory session, accessibility via a smartphone app, technical support, and reminders^d^ (n=40, with 78% men)	Regular cardiac rehabilitation for 36 weeks (weekly in-person meeting) (n=40, with 85% men)

^a^PA: physical activity.

^b^RCT: randomized controlled trial.

^c^PI: Principal Investigator.

^d^Interventions with a d superscript are the ones analyzed in this review.

^e^QoL: quality of life.

^f^GDM: gestational diabetes mellitus.

^g^ND: nondisposible.

^h^HCPs: health care professionals.

^i^T2DM: type 2 diabetes mellitus.

^j^WC: waist circumference.

^k^MS: multiple sclerosis.

^l^Adults refer to 18 years and older unless specified.

^m^HbA_1c_: hemoblogin A_1c_.

^n^DSME: diabetes self-management education.

^o^HTN: hypertension.

^p^HRQoL: health-related quality of life.

^q^COPD: chronic obstructive pulmonary disease.

^r^heiQ: health education impact questionnaire.

^s^OA: osteoarthritis.

^t^The selected article was Widmer et al, 2017 [28] and additional information about the interventions were collected from Widmer et al, 2015 [44].

^u^CV-related ED visit: cardiovascular-related emergency department visit.

### Study Population

In the selected studies, 19 included an adult population (age 18 years) [13,25,27-34,37-43] and 1 included an adolescent/adult population aged 16 years [26]. The sample size varied from 22 to 456 participants. Seventeen studies included both genders [13,25-33,35,37,39-43]. The intervention length ranged from 8 weeks to 12 months, and in 1 article, the length was not specified [34].

### Web Educational Components

The web-based interventions are summarized in Table 1.

### Features and Acceptability

The main features included in the web-based educational intervention and their acceptability are summarized in Table 2. None of these main features were identified in the studies by Noh et al [32] and Sainsury et al [26].

Only 8 studies (8/20, 40%) discussed the acceptability of the features. Acceptability was evaluated based on feedback from the users [33,36,38], their association with clinical outcomes [13,31,33,38,40,41,43], or device (eg, pedometer) adherence [43]. The features that reported positive, negative, or mixed acceptability in the studies are presented with a “+,” “−,” or “±” symbol in Table 2.

**Table 2:** Main features included in the web-based educational intervention and their acceptability.

Articles and features	Introductory session	Goal settings	Self-monitoring	Quiz	Feedback	Reminder	Online community
Bosak, 2010, United States [27]	✓^a^	✓	✓	✓	✓	x^b^	✓
Burns, 2013, Australia [42]	x	x	x	x	x	✓	x
Carolan-Olah, 2019, Australia [34]	✓	x	x	✓	x	x	x
Hansel, 2017, France [30]	x	✓	✓	x	✓	x	x
Hutchesson, 2016, Australia [36]	x	−^c^	✓	+^d^	+	x	−
Jane, 2017, Australia [37]	✓	✓	✓	x	x	x	✓
Leahey, 2014, United States [13]	✓	✓	+	x	✓	x	x
Liu, 2018, Canada [29]	x	✓	x	x	x	x	x
Morgan, 2011, Australia [38]	✓	✓	±^e^	x	±	x	−
Moy, 2016, United States [43]	x	+	✓	x	+	✓	+
Richardson, 2007, United States [33]	✓	±	+	x	+	x	x
Rothert, 2006, United States [39]	x	x	x	x	x	✓	x
Kessel, 2016 and 2012, New Zealand [35,45]	x	x	✓	✓	x	✓	x
Kerfoot, 2017, United States [31]	x	x	x	✓	✓	x	+
Tate, 2006, United States [40]	✓	✓	✓	x	+	✓	✓
Thomas, 2015, United States [41]	✓	✓	+	✓	✓	✓	x
Umpathy, 2015, Australia [25]	x	x	✓	x	✓	x	x
Widmer, 2015 and 2017, United States [28,44]	✓	x	✓	x	x	✓	x

^a^✓: Features presented in the study but without evaluation of its acceptability.

^b^x: data not available.

^c^−: features reported having negative acceptability.

^d^+: features reported having positive acceptability.

^e^±: features with mixed acceptability.

#### Introductory Session

Face-to-face introductory sessions varying from 15 to 75 min were offered in 9 of the studies [13,27,28,33,34,37,38,40,41]. Among these, the study conducted by Carolan-Olah et al [34] specified that it was offered individually, and the study conducted by Tate et al [40] mentioned that it was offered in groups of 25 participants. The format was not specified in the other studies. The purposes of these sessions were mainly to introduce the study and provide instructions about navigating the website [28,33,34,37,38,40,41]. This session also allowed the development of personal goals, teach skills (eg, food intake self-monitoring), and provide the required material (eg, printed documents or meal supplement coupons) for the intervention [13,38,40,41]. In the selected articles, no information was provided on the usefulness or acceptability of this feature.

#### Goal Setting and Self-Monitoring

Among the selected studies, goal setting (n=11) and self-monitoring (n=13) were frequently reported. The participants were able to select their goal from a predetermined area (eg, physical activity or dietary habits) [27,29,36-38] or the goal was provided by the research team at the beginning of the intervention [13,29,30,40,41,43]. The predetermined topics were chosen according to clinical guidelines [13,29,37,41] based on participants’ self-reported physical activity baseline information (eg, number of steps) [30,43] or self-reported performance from the previous week [33,46].

Three studies reported inconsistent acceptability of goal setting [33,36,43]. Participants in the study by Hutchesson et al [36] considered this feature as one of the least used. This could be related to the technical difficulty of not knowing where to find this feature. Richardson et al [33] highlighted that more structured goals were associated with a lower level of satisfaction and adherence to the intervention among participants. However, Moy et al [43] reported that the goal-setting feature might lead to higher device (eg, pedometer) use.

#### Self-Monitoring

The term self-monitoring and self-assessment are used interchangeably in 2 studies [25,35]. Studies led by Umpathy et al [25] and Kessel et al [35] mainly used the term self-assessment to describe health-related risk assessment and information tracking (eg, pain, weight, use of medication). Ten other studies [13,27,28,30,33,36-38,41,43] used the term self-monitoring and referred only to the tracking function. As most of the studies used the term self-monitoring, *self-monitoring* was employed for this review.

Among all the studies with the tracking function, 6 studies requested daily self-monitoring throughout the [13,27,28,38,41,43] intervention. Other studies requested self-monitoring for a specific period (eg, participants need to complete the self-monitoring module in 1 week before going to the other modules [30,37] or by completing the module [35]), weekly, or longer self-monitoring for specific parameters (eg, weight change) [25,33,36,38,46]. The majority of the self-monitored data were entered directly into the intervention website [13,25,27,28,30,35,38,41,43,46], and one study used a smartphone app that was not designed by the research team [36]. In the study by Hutchesson et al [36], self-monitoring was captured in a quiz format where questions allowed participants to track their weight, eating habits, and physical activity level.

The acceptability of self-monitoring was evaluated in 4 studies [13,33,38,41]. Studies found that a greater frequency of self-reporting correlated with better clinical outcomes [13,38,41], increased mindfulness in food choices [38], or higher satisfaction with the intervention [33]. However, the participants in the study conducted by Morgan et al [38] expressed that it was difficult to use this feature and to remember the food eaten. These barriers might also explain the low compliance (<50%) in this study. However, the embedded *save favorite meals* feature was reported to simplify the recording process.

#### Quiz and Feedback

Quizzes were used in 6 studies [27,31,34-36,41]. They were mainly embedded within the web-based intervention, except in the studies by Hutchesson et al [36] and Kerfoot et al [31], where the questions were sent to participants by email or via a smartphone app. In addition to being used as a tracking method [36], the quizzes had the objective of introducing the learning material [31], learning reinforcement [27,34,35], and increasing participants’ engagement [27,41]. Quizzes were included within the educational module [34,45] or sent periodically to the participants [27,31,36].

Feedback was used to reflect the progress of self-monitoring [13,25,30,33,38,40,41,43,46], the responses of the quizzes [27,31,36], and/or used as email communication with physicians [41]. In 8 of the studies, a report format was used either weekly [13,27,30,33,40,41,43,46] or periodically [38] as a follow-up to the self-monitoring data. Tate et al [40] also provided an automated weekly feedback report on the general performance of the participants for those who submitted their self-monitoring entries. In addition to summarizing the progress toward the goal [13,27,36,38,41,43], the report could also include recommendations [25,36,38,40,41], praise for achieving the goal [33,40,41], anecdotes [38], or the amount of virtual points/diamonds accumulated [36] or provide a personalized menu [30]. Among these, the use of an algorithm for generic messages or a standardized email based on the performance of each participant was used to build this report [13,25,27,33,38,40,41] and was specified in 7 of the studies. Rothert et al [39] noted the optional *buddy* feature where participants can receive email encouragement. However, no information was given on its specificity or the email content.

For feedback related to the quizzes, the correct answer and an explanation were often given immediately following the participants’ responses [27,31]. The intervention led by Kessel et al [35] used the term *interactive tasks* and *homework* for the quiz feature. In this study, the completed quizzes were discussed in the following module, but the presence or absence of feedback to the participants’ answers was not specified. Communication letters to physicians were used in 1 study and sent to the referring physician at 3 time points during the intervention [41].

The quiz feature was considered by the participants in 1 study as useful for providing information and feedback [36]. A similar observation was found in the study led by Richardson et al [33], where participants expressed their support for feedback on their step performance using a graph format. Morgan et al [38] explored the effect of the feedback feature, and the opinion was shared among participants. Some users positively highlighted its usefulness in helping people to realize their possible dietary issues, but others found that the feedback lacked personalization. In the study by Tate et al [40], the authors discussed that the feedback provided by both the automated computer program and the human counselor can lead to greater weight loss. This potential positive impact of the feedback feature on clinical outcomes was also reported by Moy et al [43].

#### Reminder

Seven studies included a reminder (eg, by email) to increase the intervention usage [35,39-42] or to recall the upload of self-monitoring data [28,38]. The frequency of sending the reminder varied between studies: weekly reminder emails to participants not using the web intervention only [41], occasional reminders to participants who did not recently log-in [28], weekly automatic reminders to all participants to upload their self-monitoring data [40,43] or the use of the intervention [35], reminder emails sent before the release of each management plan [39], or 1 reminder email midway of the intervention [42]. In addition to the email reminders, Widmer et al [28] also included reminders within the intervention to recall the completion of daily tasks and educational material. Other than reminding people participating in the intervention, Sainsbury et al [26] noted that email and text messages were used to manage participants’ progress toward the goal, but the study did not explicitly use the term reminder to qualify this function. No information was provided on the usefulness or acceptability of reminders in the selected articles.

#### Online Community

An online community was used in 7 studies [27,31,36-38,40,43]. Online communities included discussion forums [27,36,38,42,43], social media groups [37], game competitions [31], and buddy matching (optional pairing with another participant) [40]. The objectives of an online community were to increase social support between the participants [36,37,40,43], overcome barriers in behavioral change [27], answer questions [27,38,43], and/or increase a sense of competition [31]. The discussion forums were mainly operated by a research team member and divided into topics [27,36-38,43]. Jane et al [37] used a Facebook group to both deliver learning materials and encourage peer exchange. Tate et al [40] provided the option to the participants to be matched with another person and communicate through the web page. Kerfoot et al [28] used a game format to create an online community in which participants were grouped based on their geographic region and competed against each other by answering questions. A leaderboard displaying individual and team scores was used to increase the sense of competition.

Kerfoot et al [31] found that the positive change in mean hemoglobin A_1c_ among the participants was potentially related to participants’ engagement in the online community and through competition with others. Its positive effect was further supported by a correlation between patient empowerment and game engagement, reflected by the number of earned points. The benefit of using an online community was also reported in the study by Moy et al [43]. The researchers compared the number of step counts in a population with COPD between the intervention group (access to the web intervention) with a control group having only the pedometer and a self-monitoring log. The results showed that the intervention group had significantly better device adherence, which suggested the potential benefits of the included features (discussion forum, educational content, goal setting, and feedback). In addition, more than half of the participants (67/121, 55%) expressed that the online community forum helped them learn information on their chronic condition. However, the use of the discussion forum was negatively rated in a study on weight loss among men [38]. In this study, the acceptability of the feature was based on qualitative feedback collected from the participants. Users of this discussion forum considered that weight loss was a personal issue and participants were unlikely to participate in the forum. Users also expressed a preference for having more face-to-face contact with the instructor. This negative comment was also reflected in an acceptability questionnaire in a study targeting weight loss in women [36].

#### Other Features

In addition to the previously mentioned features, others were presented in the studies, such as the use of a pedometer, reward, adaptation of the website intervention for smartphones, and technical support.

A pedometer was provided by 7 studies as a component of self-monitoring to increase step counts [13,27,29,30,33,37,43].

The use of rewards was mentioned in 3 studies. A social reward included praise in a weekly report to participants who reached their goal [41] and the use of online rewards (eg, virtual diamonds) [36] indicated participants’ progress toward the goal. Only 1 study reported the use of material rewards [31], such as a US $100 certificate was given for the top 30% of participants based on their game points. It was also mentioned that the reward feature was included in the intervention led by Widmer et al [28], but no description was provided.

The adaptation of the website to mobile devices was specified in 3 studies [28,31,32].

The presence of technical support was mentioned in 3 studies. Participants could ask their questions by posting on a designated section of a discussion forum [43] via a link through the web-based program [28] or through hotline support [30]. In all instances, direct communication with a research team was restricted to technical support purposes.

### Adherence to the Intervention

Adherence to the intervention was mentioned in 15 studies (75% of the eligible studies, 15/20) using different terms (eg, engagement, use of intervention, retention rate). The rate was reported in 4 studies. The parameters used to measure adherence to the intervention are summarized in Textbox 2.

A decrease in the use of the intervention throughout the study was observed in 6 studies [26,30,36,40,41,43]. For the length of a 16-week intervention, the percentage of log-ins in the study by Hansel et al [30] decreased by one-third in the final month. Moy et al [43] reported a similar decrease in the number of log-ins with time (from 6.8 per month in the first month to 3.0 per month at 12 months). A decrease in the use of the features was also observed, such as the number of opened newsletters [36], answered quizzes [36], and the use of the discussion forum [43]. A similar decrease in the frequency of monthly log-ins was observed in the study by Tate et al [40]. Although this decrease seemed to be progressive with time, Thomas et al [41] reported that it mainly occurred midintervention, 3 months from the beginning. Hutchesson et al [36] also observed that some features (eg, discussion forum and goal settings) had poor usage throughout the intervention and Morgan et al [38] reported that <50% of their participants complied with self-monitoring instructions. However, based on the general use of the intervention (eg, 7 weeks of submission of self-reporting data and weekly log-ins during the 3 months of the intervention), Morgan et al [38] qualified a retention rate of 41% as high. The term retention rate was also used by Sainsbury et al [26] and was measured with the use of the intervention. It was shown that 49.5% of the participants completed 4 of the 5 learning modules, but the authors considered this as a poor retention rate. Kessel et al [35] related the high dropout level (9/20, only 45% of the participants completed the intervention) to the absence of individual support, lack of feedback, and technical challenges. Bosak et al [27] explained that participants with better adherence had increased self-efficacy, but no additional information was provided.

**Parameters used to evaluate adherence to the intervention and the methods of measurement.**

**Log-in to the intervention**

- Track of the total frequency of the log-in [30,32,36,40,42]
- Average log-in per participant [42]
- Average log-in per week per person [13]
- Average log-in per month per person [43]
- Number of weeks with at least one log-in [41]
- Total number of visits [42]

**Exploration of the learning content**

- Number of participants completed at least 4 out of the 5 modules [26]
- Number of lessons viewed [13]
- Number of participants who completed none, half, or all the 8 sessions [35]
- Mean number of sessions completed [35]

**Upload of the self-monitoring data**

- Total frequency of self-monitoring [13,33,38]
- Number of weeks having self-monitoring values at least 5 of the 7 days [41]
- Frequency of weekly web-based diary submission [40]

**Use of other features**

- Use of the discussion forum [36,43]
- Use of the discussion forum [36,43]
- Number of answered questions [31]
- Number of points earned during the game [31]
- Completion of quizzes, number of email newsletters opened, and smartphone app downloads [36]

**Visit duration**

- Total duration of viewing [42]
- Average viewing time by participant [42]

## Discussion

### Principal Findings

This systematic review highlights the use of specific features in the design of web-based self-guided interventions for people with chronic health conditions and reports on the evaluation of their acceptability. Previous researchers have investigated the importance of features included in guided web-based interventions for people with chronic diseases on their success rate (eg, adherence to the intervention and transfer of health-related information) [11,12]. However, limited data were found on the functionalities of self-guided web-based educational interventions. In-person and one-on-one interactions with an HCP might increase the adherence and use of a web-based intervention [47] but that can also increase the cost of the intervention [13]. Therefore, it is important to investigate the characteristics of web-based interventions. This review demonstrated that goal setting, self-monitoring, and feedback were the most common features. The acceptability of the different features was measured based on the comments collected from users, their influence on clinical outcomes, or device (eg, pedometer) adherence. The use of personalized features with feedback (eg, quizzes) was positively reported. The negative acceptability of the features was mainly related to technical issues and the choice of discussion topics for the intervention. This review also showed that the evaluation of adherence to the intervention was inconsistent among the studies, which limited comparison. A clear definition and measurement of adherence to web-based interventions is lacking.

### Categorization of Features

Our review identified 7 features that were most commonly included in the selected studies (Table 2). Other features such as the use of a pedometer, rewards, adaptation of the website intervention for smartphones, and technical support were also observed but less frequently used. On the basis of the results of this paper, we categorized the included features under the following 3 categories: personalization, interaction, and support. Personalization refers to a function tailored to the individual needs of each participant and can be changed throughout the intervention based on the user’s experience and progress [12]. Goal setting and self-monitoring have this characteristic by adjusting to the needs and progress of the user. The interactive features facilitated the engagement of the participants, increased learning retention [36], and provided a sense of community [31]. These characteristics were found in features such as quizzes, feedback, reminders, and online communities. They allowed an interaction between the intervention and participants and encouraged the users to return to the intervention [27,34,36]. Feedback and reward features correspond to both categories by personalizing the feedback report and varying the amount of rewards or type of written encouragement given to the participants based on the individual’s progress [31,36]. Other features not included in these 2 categories were providing support and reducing the technical barriers of the intervention.

### Importance of Evaluating the Features

Web-based educational interventions have been shown to be cost-effective compared with traditional face-to-face formats [48-51] and can reduce the production of physical materials (eg, printed documents) [52]. However, the cost related to the development of web-based educational interventions is still significant [52]. Creation of web-based educational modules can be classified into 3 levels: (1) basic content with text, graphics, simple audio, video, and test questions, (2) level 1 content with 25% interactive content (exercise, audio, video, and animations), and (3) level 2 content with highly interactive features (eg, adding game, avatars, custom interactions, and competitions) [53]. According to a study published in 2010, the average number of working hours to produce 1 hour of finished training associated with each of these levels is at least 79, 184, and 490 hours, respectively [53], and the average cost in US dollars is $10,054, $18,583, and $50,371, respectively [53]. Other factors such as the addition of new content and interactive features will further increase the cost [53]. Therefore, it is important to consider the choice of the features and their evaluation to minimize the cost and distribute the financial resources effectively. Our systematic review highlights that features are not frequently evaluated, with only 8 studies (8/20, 40%) reporting on the evaluation of some of the features used. In addition, the negative acceptability of a feature on the user’s experience, clinical outcomes, or device adherence was shown to be related to a lack of responding to the population’s needs, low human contact, and technical difficulties.

### Factors Impacting the Acceptability of a Feature

#### Lack of Responding to the Population’s Needs

A previous systematic review investigating features to be included in a commercial smartphone app for people with type 1 diabetes highlights the importance of integrating features related to personalization and patient empowerment for optimal disease self-management [54]. Similar to this study, our review showed the benefits of these groups of features [36,38]. For instance, the self-monitoring feature showed positive acceptability for the user’s experience, clinical outcomes, or device adherence. Participants in a weight loss intervention conducted by Morgan et al [38] expressed that the self-monitoring features helped to increase mindfulness of their dietary choices. The participants also liked the *save favorite meals* option, which was associated with their eating habits and facilitated their diet entries [38]. Another feature that can increase patient empowerment is feedback, but it was found to lack personalization. Being able to effectively provide information [36] and improve behaviors [38] are some of the benefits of providing feedback through self-monitoring and quizzes. However, the use of a generic message was criticized by some participants and they expressed a preference for having more personalized communication [38]. This evidence shows the potential benefits of these features and highlights the necessity of adapting them to patients’ needs.

Indeed, the effectiveness of a feature can only be maximized when there is a deep understanding of the targeted population’s needs [15,38]. For example, peer support is often identified as an essential component in web-based interventions across different areas of health care [55-58], but its use should be based on the specific population’s preferences. Kerfoot et al [31] and Moy et al [43] found a positive correlation between participants’ engagement, learning, and use of an online community. However, men in a weight loss study also expressed their resistance in using the discussion forum mainly because of the personal nature of the topic and they preferred to have face-to-face contact with their instructor [38]. Similar feedback was also reported in a weight loss study in women [36]. As the interest and needs of patients vary with different types of chronic diseases, the topics involved in these discussion forums should also be based on the interests of the population group being targeted. For instance, Lanoye et al [59] found the importance of discussing the stigma and peer pressure related to obesity within a young adult population, whereas Cook et al [60] found that emotional support and use of medication are priorities in an older population with obesity. Therefore, the demographic background [11,61,62] and type of chronic diseases [7] are all factors potentially influencing the acceptability of a feature and should be considered when designing and evaluating web-based interventions.

#### Low Human Contact

In addition to the lack of responding to the population's needs, the frequency of human contact was another element mentioned in the selected studies that could interfere with the acceptability of a feature [36]. Hutchesson et al [36] suggested that the low level of human contact in their weight loss intervention could have been a reason for the low usage of the discussion forum. Leahey et al [13] verified this hypothesis in their study on weight loss by adding a face-to-face component to their web-based intervention; however, it was shown that improved clinical outcomes also resulted in a higher monetary cost. Kessel et al [35] also mentioned that having human contact (eg, telephone support) might lead to a higher engagement with the intervention. Therefore, a greater in-person or one-on-one consultation with an HCP in the intervention has the potential to increase its efficacy, but the cost should also be considered. As the goal of this systematic review is to investigate the features presented in self-guided web-based interventions, with the primary inclusion criteria of the studies being the absence of face-to-face contact, it would be contradictory to suggest the addition of a face-to-face component for an intervention. However, having patient moderators implicated in the intervention can be a potential solution for this barrier [63].

Moderators have the role of being the *housekeeper* of the discussion forum. They adopt an objective point of view by balancing the opinions of different sources in a respective environment. It also acts as a conversation stimulator, conflict resolver, feedback provider, and discussion supporter [63,64]. Previous studies highlighted the importance of their role by showing that participants can develop an attachment with community moderators and that their departure can lead to cessation in the use of the forum among some participants [65]. Having HCPs and peer moderators will combine the expertise for the delivery of web-based interventions [12]. As the use of the intervention is also associated with its impact (eg, on clinical outcomes or behavioral change) [12], it is important to be able to define and measure the level of adherence [12]. Adherence can be associated with factors such as chronic health conditions [26,42], study design, and inclusion of a variety of features [12,66]. In our review, the eligible studies reported different ways of measuring adherence to the interventions (eg, log-ins to the intervention [42], exploration of the learning content [13], and uploading of the self-monitoring data [41]) using different terms (eg, engagement [36], retention rate [38]), and none of them defined the effective engagement or intended usage of the intervention.

#### Technical Difficulties

Technical barriers were a third reason for the lower acceptability of a feature. Users in the weight loss trial conducted by Morgan et al [38] expressed that despite an improvement in behavioral changes related to the use of self-monitoring, the difficulty in tracking their food decreased their use of the intervention. Hutchesson et al [36] also suggested that the lack of usage of the goal-setting feature might be related to the difficulty in finding this feature in the intervention. This low usage was attributed to technical issues, and was previously reported in the literature [14]. The action planning feature usage was reported as relatively low in a study of people with type 2 diabetes conducted by Glasgow et al [14], and this could be related to navigational difficulties. These observations highlight the importance of simplifying the intervention navigation and including technical support features (eg, introductory session), providing contact information of the research team, and technology usage learning to help decrease these barriers [67].

#### Adherence and Future Direction

Intended usage is estimated by the developers and refers to the usage level needed to have the maximum benefit from the intervention (eg, clinical outcomes), and defining the intended usage would allow for standardization in the calculation of adherence [12]. Although Kelders et al [12] used the term intended usage, others adopted the term effective engagement [68,69], defined as "sufficient engagement with the intervention to achieve intended outcomes" [69]. As both terminologies focused on the identification of the parameters and the related minimum threshold that can have an impact on the intended behavior [12,68,69], these terms were used interchangeably.

Effective engagement should reflect the multidimension of the intervention in relation to the primary outcome, and both objective and subjective measurements should be evaluated [70]. The back-ended intervention usage data are considered an objective measurement [70] and can be assessed by using the Analyzing and Measuring Usage and Engagement Data framework [68]. This framework is designed for web-based interventions and can be used during the intervention development phase or after data collection. It contains 3 stages, and each stage is guided by a checklist of generic questions. In stage 1, the usage of data is classified into 3 categories: intervention characteristics (eg, architecture and content), accrued data (eg, data collected during the use of the intervention), and contextual data (eg, factors influencing the use of the intervention). Stage 2 consists of the selection of meaningful measures of usage and generation of research questions related to the primary outcome, usage data collected, and characteristics of the target population (eg, a web-based intervention focusing on the reduction of hospital visits can have “Will the number of content views be associated with hospital visits?” as a research question [68]). The final stage focuses on the selection of analytical tools and data preparation. A plan of analyses can then be conceived if the intervention is in the developmental phase or the analyses can be performed if data have already been collected [68]. In addition to the usage data, qualitative analysis (eg, with a semistructured interview or focus group) should be performed and combined with the quantitative methods [70] to reflect participants’ experiences. The threshold of effective engagement found with the combination of these 2 methods can then be compared with the actual intervention usage of each participant. Those who failed to reach this threshold will then be categorized as nonadherent to the intervention. Therefore, adherence to the intervention and its cutoff should only be defined after data collection is completed and a proof-of-concept or pilot study is recommended for testing [71].

### Limitations

Our systematic review had some limitations. The search terms were selected based on MeSH terms; however, other important keywords could have been included. Exclusion of these important keywords might decrease the level of comprehensiveness of the search results. All the qualitative analyses were based on the content of the articles; the omission of information within the published articles might have led to a different interpretation of the results. For example, authors might only have listed the major features in their intervention instead of providing a complete list of all the available features. Only 8 studies (8/20, 40%) reported the acceptability of the features on the clinical outcome, users’ experience, or device adherence, which is a limitation for extrapolating the conclusions of the interventions. The articles included in this review were only selected from 3 databases, limited to published or in-press articles in English and French. In addition, to ensure a higher level of effectiveness in the results, this review also excluded self-guided interventions having individual contact between participants and research professionals during the study for reasons other than technical support or introductory sessions. Therefore, the results of this review might have limited external validity and cannot be applied to all web-based self-guided interventions or specific to any of the selected disease categories.

### Conclusions

In conclusion, this systematic review investigated features included in 20 self-guided web-based educational interventions focusing on the self-management of chronic health conditions. It demonstrated the positive implication of specific features related to personalization and interactivity in the interventions on clinical outcomes, users’ experience, or device adherence. However, only a few studies reported the acceptability of the included features; therefore, future research is needed to gain a greater understanding of the roles that each feature plays on the use of web-based interventions. The results of this systematic review provide evidence on the choice and implementation of specific features for future web-based health education interventions, highlighting the importance of understanding the needs of the target population and the need to incorporate more human contact and reducing technical barriers for the effectiveness of self-guided web-based interventions. Moreover, this study also found poor consensus related to the definitions and measurements of adherence in self-guided interventions used to target chronic health conditions. A method for evaluating the level of adherence is proposed in this review but requires future studies for its validation.

## Appendix

Multimedia Appendix 1 Keywords used for the article searches.

## Abbreviations

COPD: chronic obstructive pulmonary disease
e-counseling: electronic counseling
EMBASE: Excerpta Medica dataBASE
GDM: gestational diabetes mellitus
HCP: health care professional
MeSH: Medical Subject Headings
