# Supplementary material for: Understanding Self-Guided Web-Based Educational Interventions for Patients With Chronic Health Conditions: Systematic Review of Intervention Features and Adherence
Source: J Med Internet Res. 2020 Aug 13;22(8):e18355. doi: 10.2196/18355 (PMC7473470; doi:10.2196/18355)
Supplement: Multimedia Appendix 1 [file jmir_v22i8e18355_app1.docx]

## Multimedia Appendix 1. Keywords used for the article searches.

The searches were performed on June 15 2020 on PubMed, Cochrane Library and Embase.

### Search on PubMed

The search was applied to *Title/Abstract*, limited to Full text, English and French, years 2005 to 2020.

The following site was used:

<https://pubmed.ncbi.nlm.nih.gov/>

Each of the following paragraphs represents one search entry:

(“social media” OR Internet OR “ web based” OR web OR online) AND (“distance education” OR education OR “patient education” OR teaching) AND (learning OR intervention OR treatment OR program OR “Program development” OR platform) AND (“pediatric obesity” OR “abdominal obesity” OR “morbid obesity” OR “obesity management” OR “Abdominal obesity” OR “metabolic syndrome” OR “overweight” OR “metabolic syndrome” OR “weight reduction program”)

(“social media” OR Internet OR “ web based” OR web OR online) AND (“distance education” OR education OR “patient education” OR teaching) AND (learning OR intervention OR treatment OR program OR “Program development” OR platform) AND (CVD OR hypertension OR diabetes OR “diabetes mellitus” OR “diabetes insipidus” OR “gestational diabetes” OR “type 2 diabetes mellitus” OR “type 1 diabetes mellitus” OR “Juvenile diabetes” OR “heart failure” OR atherosclerosis OR dyslipidemia OR “Cardiovascular disease”)

(“social media” OR Internet OR “ web based” OR web OR online) AND (“distance education” OR education OR “patient education” OR teaching) AND (learning OR intervention OR treatment OR program OR “Program development” OR platform) AND (IBD OR “inflammatory bowel disease” or “crohn disease” or “ulcerative colitis”)

(“social media” OR Internet OR “ web based” OR web OR online) AND (“distance education” OR education OR “patient education” OR teaching) AND (learning OR intervention OR treatment OR program OR “Program development” OR platform) AND (“respiratory disease” or “respiratory tract disease” or “respiratory disorder” or “asthma” or “chronic respiratory disease” or “copd” or “chronic obstructive pulmonary disease”)

(“social media” OR Internet OR “ web based” OR web OR online) AND (“distance education” OR education OR “patient education” OR teaching) AND (learning OR intervention OR treatment OR program OR “Program development” OR platform) AND (celiac)

(“social media” OR Internet OR “ web based” OR web OR online) AND (“distance education” OR education OR “patient education” OR teaching) AND (learning OR intervention OR treatment OR program OR “Program development” OR platform) AND (epilepsy)

(“social media” OR Internet OR “ web based” OR web OR online) AND (“distance education” OR education OR “patient education” OR teaching) AND (learning OR intervention OR treatment OR program OR “Program development” OR platform) AND (“chronic kidney disease” or “chronic renal insufficiency” or “kidney disease” or “chronic kidney failure” or “diabetic nephropathies” or “esrd” or “end stage renal disease” or “nephritis”)

(“social media” OR Internet OR “ web based” OR web OR online) AND (“distance education” OR education OR “patient education” OR teaching) AND (learning OR intervention OR treatment OR program OR “Program development” OR platform) AND (arthritis)

(“social media” OR Internet OR “ web based” OR web OR online) AND (“distance education” OR education OR “patient education” OR teaching) AND (learning OR intervention OR treatment OR program OR “Program development” OR platform) AND (“multiple sclerosis”)

### Search on Cochrane Library

The searches on Cochrane Library used the same keywords as in PubMed.

The keywords were searched for *Title Abstract Keyword*, years 2015 to 2020, Trials, English.

(No French language was available because no French abstract was found on Cochrane Library.)

It was done on the following website: <https://www.cochranelibrary.com/search>

### Search on Embase

The search was applied to *Title* or *Abstract*, limited to Full text, English and French, year 2005 to current, exclude Medline Journals, human, article and article in-press.

Each of the following paragraphs represents one search entry:

(social media OR Internet OR web based OR web OR online) AND (distance education OR education OR patient education OR teaching) AND (learning OR intervention OR treatment OR program OR Program development OR platform) AND (pediatric obesity OR abdominal obesity OR morbid obesity OR obesity management OR Abdominal obesity OR metabolic syndrome OR overweight OR metabolic syndrome OR weight reduction program)

(social media OR Internet OR web based OR web OR online) AND (distance education OR education OR patient education OR teaching) AND (learning OR intervention OR treatment OR program OR Program development OR platform) AND (CVD OR hypertension OR diabetes OR diabetes mellitus OR diabetes insipidus OR gestational diabetes OR type 2 diabetes mellitus OR type 1 diabetes mellitus OR Juvenile diabetes OR heart failure OR atherosclerosis OR dyslipidemia OR Cardiovascular disease)

(social media OR Internet OR web based OR web OR online) AND (distance education OR education OR patient education OR teaching) AND (learning OR intervention OR treatment OR program OR Program development OR platform) AND (IBD OR inflammatory bowel disease or crohn disease or ulcerative colitis)

(social media OR Internet OR web based OR web OR online) AND (distance education OR education OR patient education OR teaching) AND (learning OR intervention OR treatment OR program OR Program development OR platform) AND (respiratory disease or respiratory tract disease or respiratory disorder or asthma or chronic respiratory disease or copd or chronic obstructive pulmonary disease)

(social media OR Internet OR web based OR web OR online) AND (distance education OR education OR patient education OR teaching) AND (learning OR intervention OR treatment OR program OR Program development OR platform) AND (celiac)

(social media OR Internet OR web based OR web OR online) AND (distance education OR education OR patient education OR teaching) AND (learning OR intervention OR treatment OR program OR Program development OR platform) AND (epilepsy)

(social media OR Internet OR web based OR web OR online) AND (distance education OR education OR patient education OR teaching) AND (learning OR intervention OR treatment OR program OR Program development OR platform) AND (chronic kidney disease or chronic renal insufficiency or kidney disease or chronic kidney failure or diabetic nephropathies or esrd or end stage renal disease or nephritis)

(social media OR Internet OR web based OR web OR online) AND (distance education OR education OR patient education OR teaching) AND (learning OR intervention OR treatment OR program OR Program development OR platform) AND (multiple sclerosis)

(social media OR Internet OR web based OR web OR online) AND (distance education OR education OR patient education OR teaching) AND (learning OR intervention OR treatment OR program OR Program development OR platform) AND (arthritis)

### D) The reference list of the selected articles was also searched for potential articles
